# Supplementary material for: The C. elegans gustatory receptor homolog LITE-1 is a chemoreceptor required for diacetyl avoidance
Source: bioRxiv. 2026 Oct 6:2025.04.20.649642. Preprint. [Version 3] doi: 10.1101/2025.04.20.649642 (PMC13419714; doi:10.1101/2025.04.20.649642)
Supplement: Supplementary file 1 [file NIHPP2025.04.20.649642v3-supplement-1.pdf]

bioRxiv preprint doi: https://doi.org/10.1101/2025.04.20.649642; this version posted October 6, 2026. The copyright holder for this preprint (which was not certified by peer review) is the author/funder, who has granted bioRxiv a license to display the preprint in perpetuity. It is made available under a CC-BY 4.0 International license.

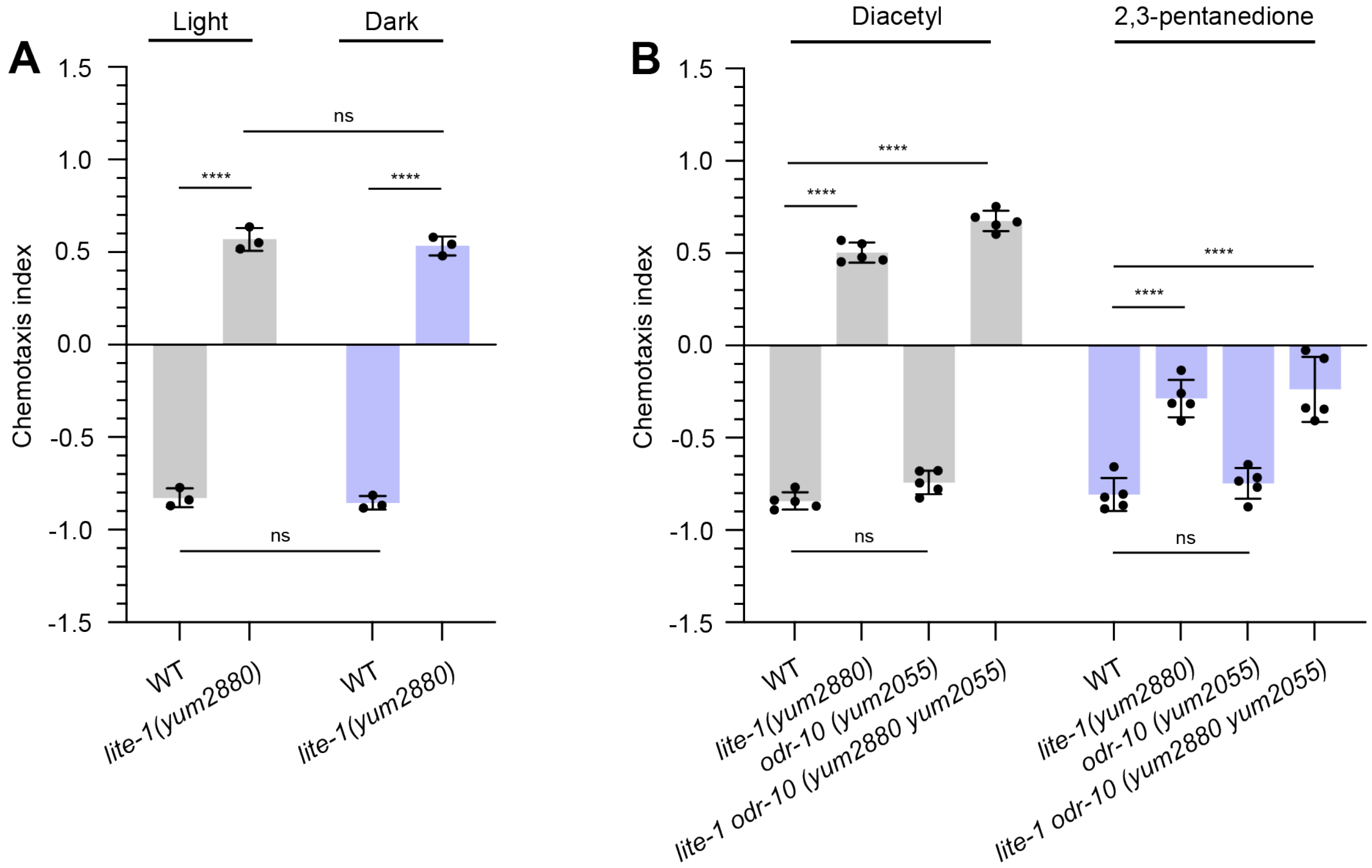

Supplementary Figure 1: (A) Chemotaxis index of wild-type and *lite-1 (yum2880)* animals with undiluted diacetyl in the presence and absence of ambience light. P-values are from unpaired two-tailed t-tests, n=3 biological replicates, each an average of 3-4 plates. (B) Chemotaxis index of wild-type, *lite-1 (yum2880), odr-10 (yum2055) and lite-1odr-10 (yum2880; yum2055)* with undiluted diacetyl or 2,3-pentannedione. P-values are from unpaired two-tailed t-tests, n=5 biological replicates, each an average of 3-4 plates.

bioRxiv preprint doi: https://doi.org/10.1101/2025.04.20.649642; this version posted October 6, 2026. The copyright holder for this preprint (which was not certified by peer review) is the author/funder, who has granted bioRxiv a license to display the preprint in perpetuity. It is made available under a CC-BY 4.0 International license.

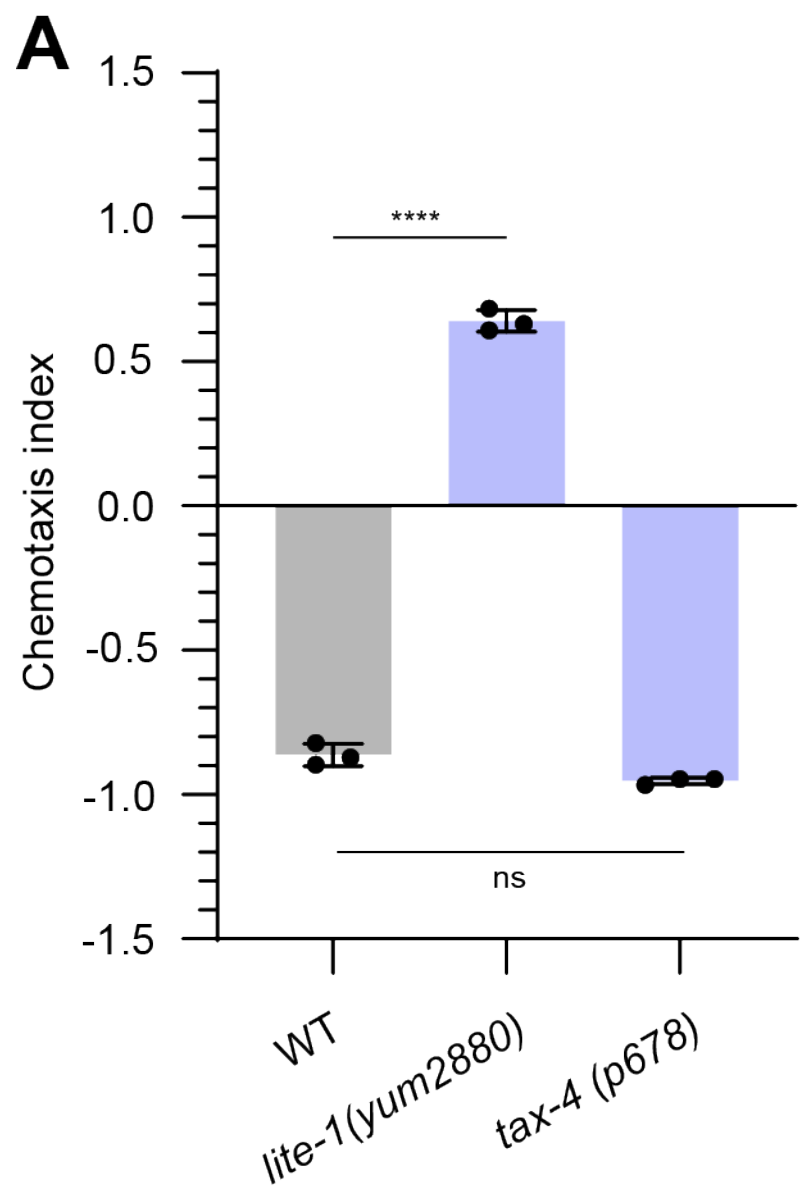

Supplementary Figure 2: Chemotaxis index of wild-type and *tax-4 (p678)* animals with undiluted diacetyl. P-values are from unpaired two-tailed t-tests, n=3 biological replicates, each an average of 4 plates.

bioRxiv preprint doi: https://doi.org/10.1101/2025.04.20.649642; this version posted October 6, 2026. The copyright holder for this preprint (which was not certified by peer review) is the author/funder, who has granted bioRxiv a license to display the preprint in perpetuity. It is made available under a CC-BY 4.0 International license.

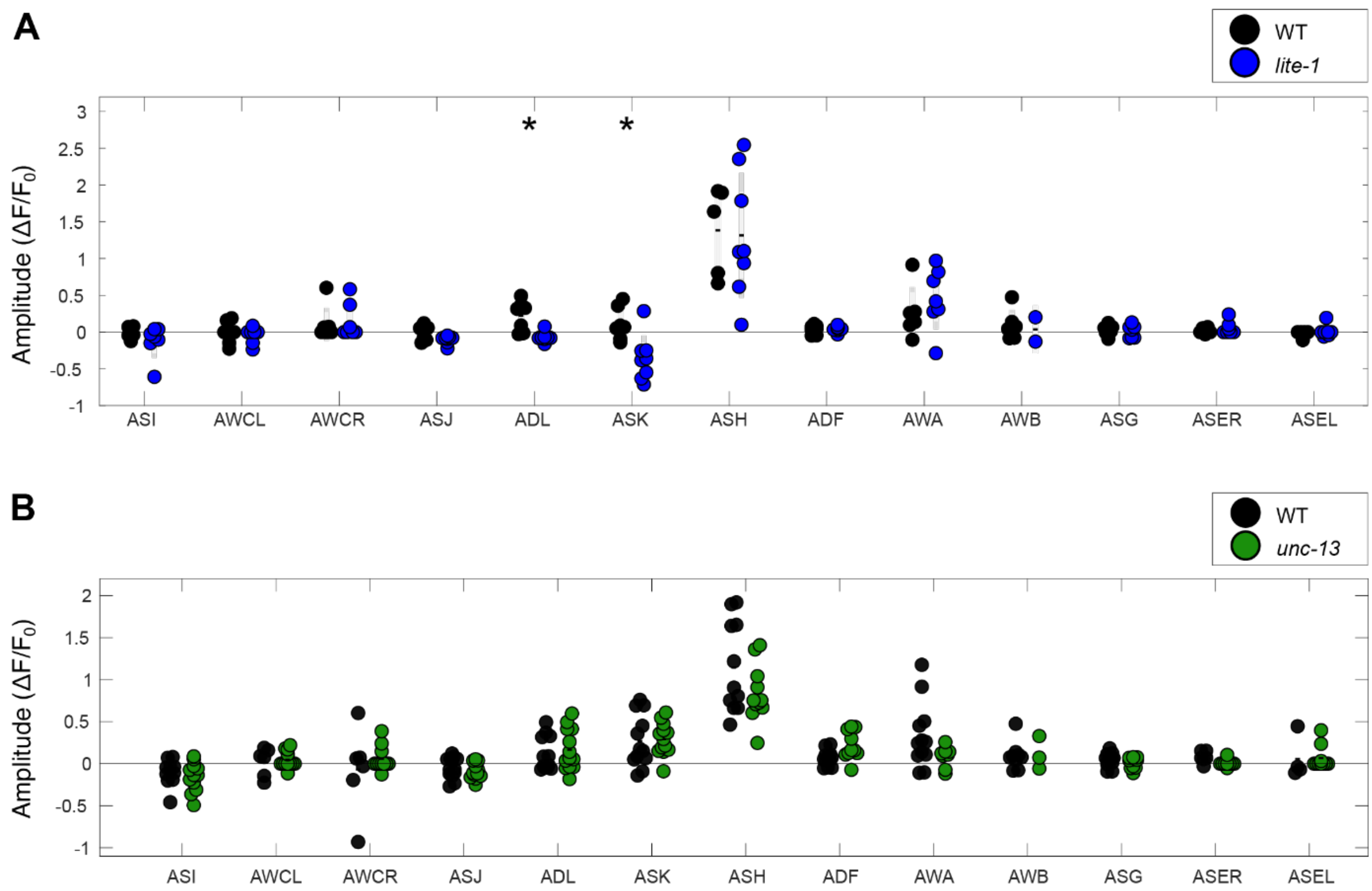

Supplementary Figure 3: Scatter plot of calcium imaging responses across 11 pairs of sensory neurons following a pulse of diacetyl. (A) ADL and ASK neurons show significant differences in activity in the presence versus absence of LITE-1. (B) No significant differences in neuronal activity between wild-type and *unc-13* mutants across the different sensory neuron pairs. P-values were calculated using two-sided t-test with FDR correction, *$p < 0.01$.

bioRxiv preprint doi: https://doi.org/10.1101/2025.04.20.649642; this version posted October 6, 2026. The copyright holder for this preprint (which was not certified by peer review) is the author/funder, who has granted bioRxiv a license to display the preprint in perpetuity. It is made available under a CC-BY 4.0 International license.

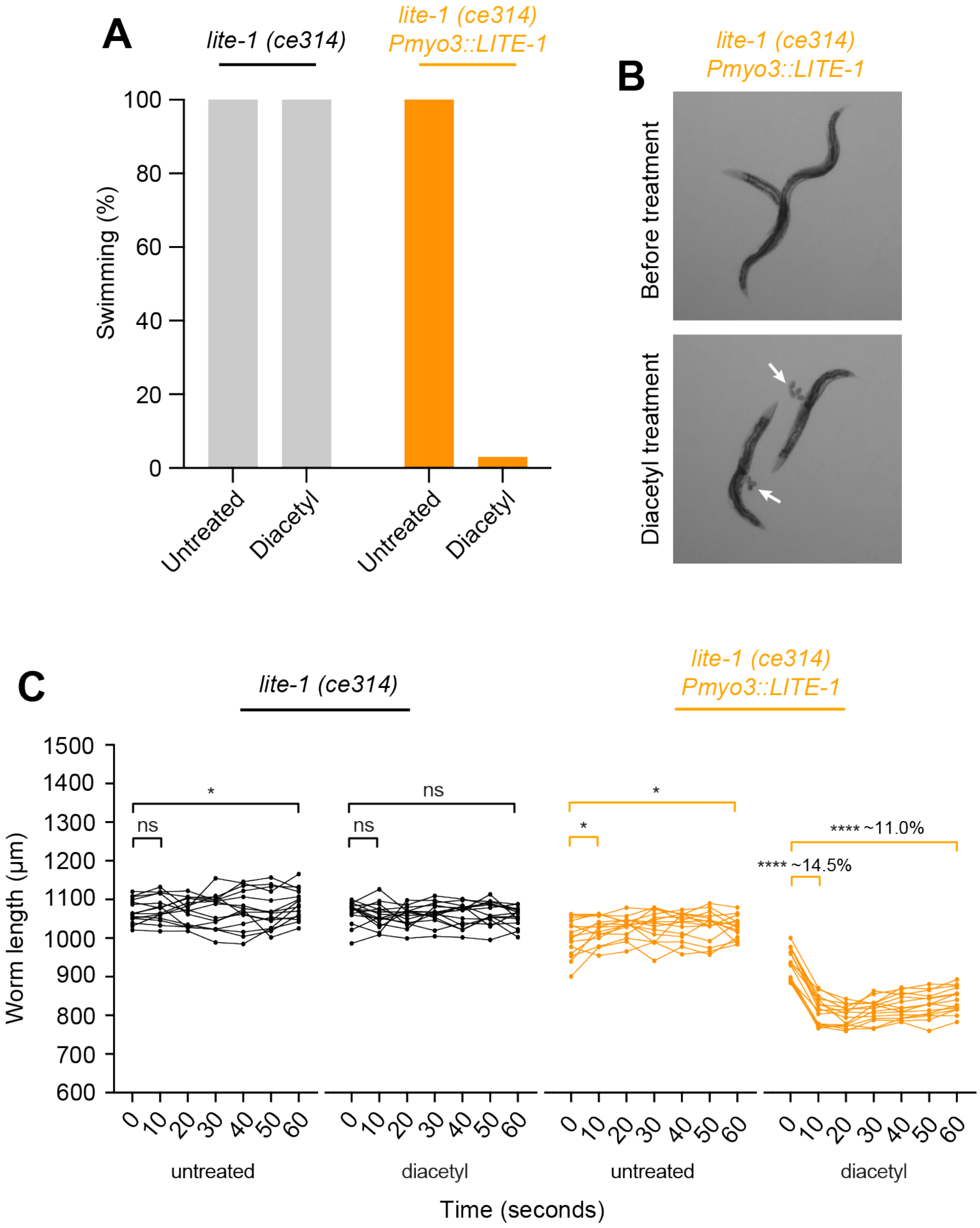

Supplementary Figure 4: (A) Percent of worms that are swimming at ten seconds after exposure to buffer containing diacetyl (*lite-1 (ce314)*, left) whereas worms expressing LITE-1 in body-wall muscles (*lite-1 (ce314) Pmyo-3::*LITE-1, right) are mostly paralysed when treated with diacetyl. (B) Image of *lite-1 (ce314) Pmyo-3::*LITE-1 animals that ejected eggs after ten seconds of diacetyl treatment (white arrows). (C) Worm body length over time after treatment. *lite-1 (ce314)* worms expressing LITE-1 in body wall muscles show a significant contraction in response to diacetyl (right; insert showing the average contraction). P-values from paired two-tailed t-test, n ≥ 15 worms.

bioRxiv preprint doi: https://doi.org/10.1101/2025.04.20.649642; this version posted October 6, 2026. The copyright holder for this preprint (which was not certified by peer review) is the author/funder, who has granted bioRxiv a license to display the preprint in perpetuity. It is made available under a CC-BY 4.0 International license.

DyamicBind			Gnina		
Ligand	Affinity (-log(IC_{50}))	Affinity Kd (μM)	Affinity (kcal/mol)	CNN pose score	CNN affinity
diacetyl	7.11	0.078	-3.82	0.832	3.699
2-butanone	6.75	0.178	-3.42	0.862	3.607
2,3-pentanedione	5.93	1.175	-4.03	0.859	4.182
1-octanone	5.86	1.380	-1.24	0.668	5.25
benzaldehyde	5.70	1.995	-5.52	0.829	4.105
isoamyl alcohol	5.46	3.467	-4.05	0.889	3.72
2-nonanone	4.73	18.621	-5.26	0.935	4.80
2,4,5-trimethylthiazole	3.13	741.310	-3.58	0.565	3.34

Supplementary Figure 5: DynamicBind and Gninna predicted ligand binding affinities of different odorants to the putative LITE-1 binding pocket. For Dynamicbind, affinity is reported as -log(IC_{50}), where higher values indicate stronger predicted binding and the corresponding Kd values(μM) were converted from -log(IC_{50}), with lower values indicating stronger binding. For Gnina, affinity is reported as kcal/mol where higher values indicate higher binding interactions.

bioRxiv preprint doi: https://doi.org/10.1101/2025.04.20.649642; this version posted October 6, 2026. The copyright holder for this preprint (which was not certified by peer review) is the author/funder, who has granted bioRxiv a license to display the preprint in perpetuity. It is made available under a CC-BY 4.0 International license.

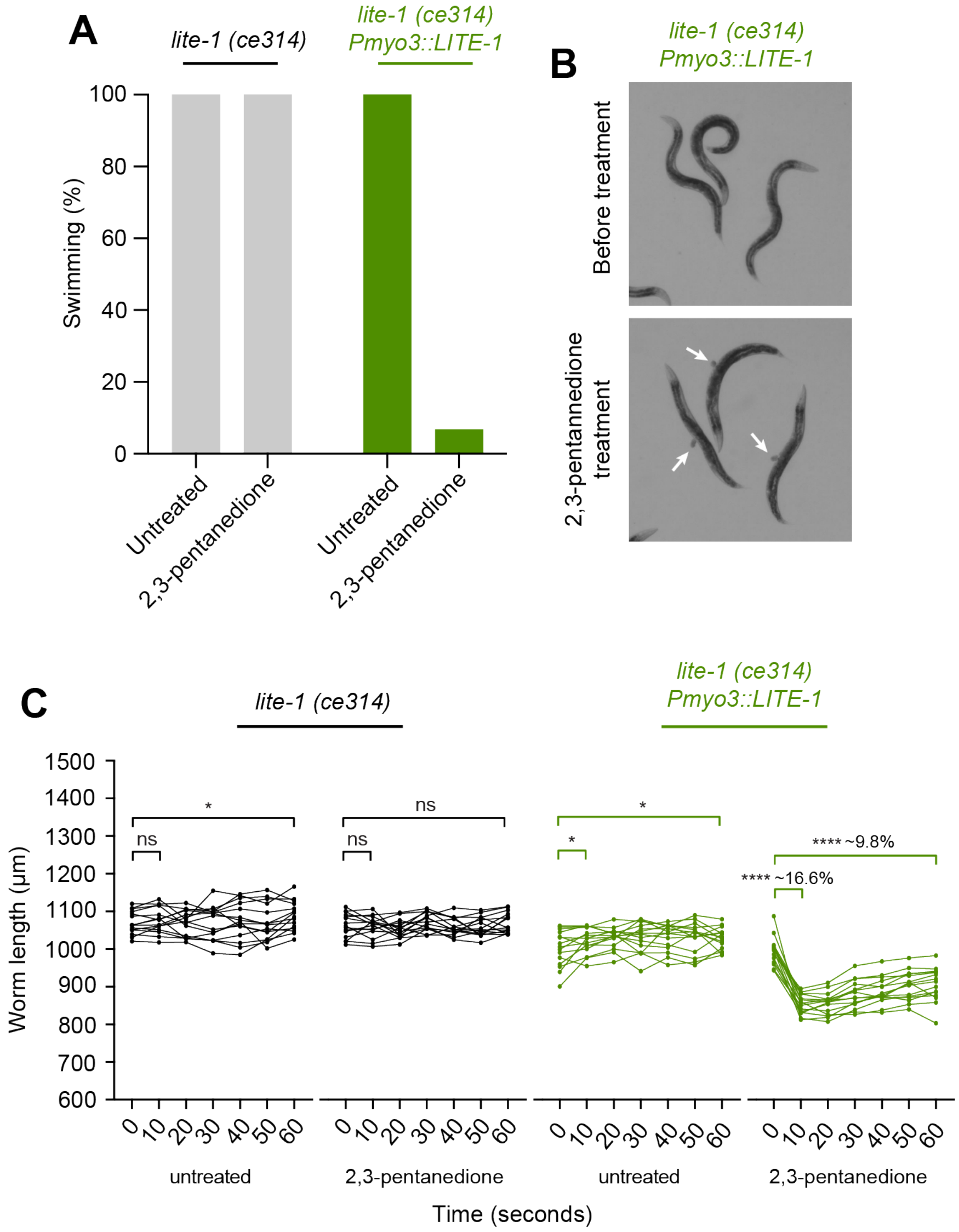

Supplementary Figure 6: (A) Percent of worms that are swimming at ten seconds after exposure to buffer containing 2,3-pentanedione (*lite-1 (ce314)*, left) whereas worms expressing LITE-1 in body-wall muscles (*lite-1 (ce314) Pmyo-3::*LITE-1, right) are mostly paralysed when treated with 2,3-pentanedione. (B) Image of *lite-1 (ce314) Pmyo-3::*LITE-1 animals that ejected eggs after ten seconds of 2,3-pentanedione treatment (white arrows). (C) Worm body length over time after treatment. *lite-1 (ce314)* worms expressing LITE-1 in body-wall muscles show a significant contraction in response to 2,3-pentanedione (right; insert showing the average contraction). P-values from paired two-tailed t-test, n ≥ 15 worms.

bioRxiv preprint doi: https://doi.org/10.1101/2025.04.20.649642; this version posted October 6, 2026. The copyright holder for this preprint (which was not certified by peer review) is the author/funder, who has granted bioRxiv a license to display the preprint in perpetuity. It is made available under a CC-BY 4.0 International license.

Supplementary video 1: Chemotaxis assay without sodium azide showing avoidance of high concentration off diacetyl by wild-type animals. Wild-type animals were recorded over 1 h following addition of diacetyl and control to the chemotaxis plate. + indicates the position where 5 µl of undiluted diacetyl was spotted, which – indicates the position where 5 µl of ethanol was spotted (https://doi.org/10.6084/m9.figshare.32745054).

Supplementary video 2: Chemotaxis assay without sodium azide showing avoidance of high concentration off diacetyl by *lite-1* mutant animals. *lite-1* mutant animals were recorded over 1 h following addition of diacetyl and control to the chemotaxis plate. + indicates the position where 5 µl of undiluted diacetyl was spotted, which – indicates the position where 5 µl of ethanol was spotted (https://doi.org/10.6084/m9.figshare.32745054).
